# Tension Pneumoperitoneum Caused by Obstipation

**DOI:** 10.5811/westjem.2015.6.25283

**Published:** 2015-10-20

**Authors:** Daniel Miller

**Affiliations:** University of Iowa Hospitals and Clinics, Department of Emergency Medicine, Iowa City, Iowa

## Abstract

Emergency physicians are often required to evaluate and treat undifferentiated patients suffering acute hemodynamic compromise (AHC). It is helpful to apply a structured approach based on a differential diagnosis including all causes of AHC that can be identified and treated during a primary assessment. Tension pneumoperitoneum (TP) is an uncommon condition with the potential to be rapidly fatal. It is amenable to prompt diagnosis and stabilization in the emergency department. We present a case of a 16-year-old boy with TP to demonstrate how TP should be incorporated into a differential diagnosis when evaluating an undifferentiated patient with AHC.

## CASE

**Figure 1:**
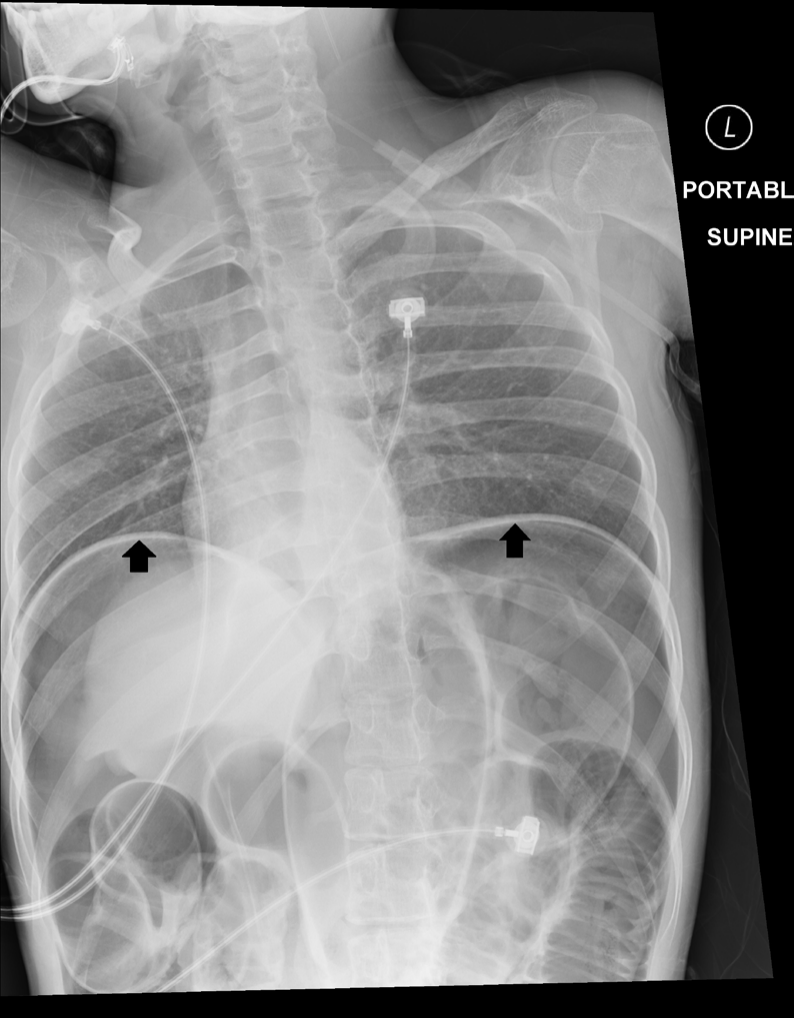
Initial chest x-ray demonstrating elevated diaphragm (black arrows).

**Figure 2:**
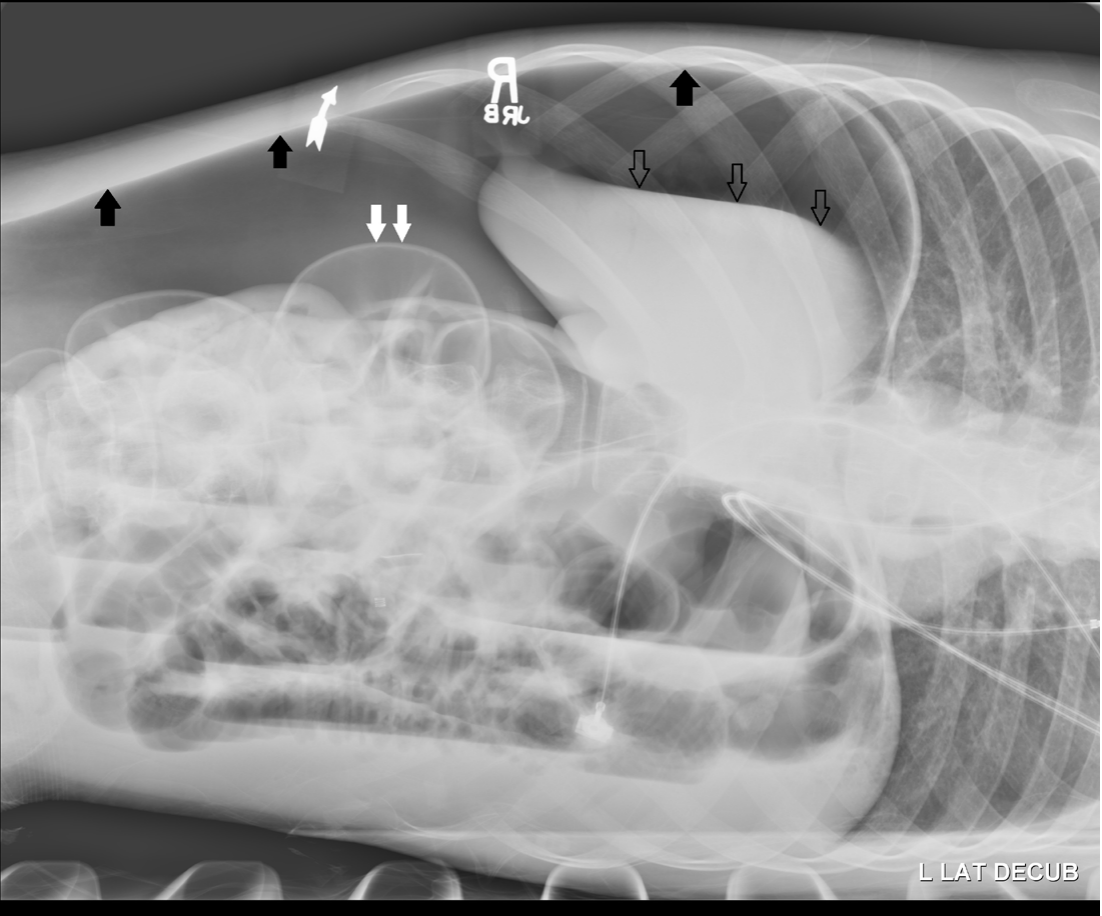
Initial abdominal film, in left lateral decubitus position demonstrating large amount of free air (black arrows), dilated loops of bowel (double white arrows) and inferior and medial displacement of the liver (“saddle bag sign” [triple clear arrows]).

A 16-year-old Hispanic male presented to our emergency department (ED) with difficulty breathing that his mother first noted on the previous night. He had a history of muscular dystrophy (MD), frequent seizures and severe developmental delay causing him to be non-verbal at baseline. He also had a long-standing history of constipation. His mother had not noted any new symptoms until the previous night when he appeared to develop difficulty breathing. Upon arrival to the ED he was found to be in respiratory distress. His vital signs were significant for the following: heart rate 144 beats/minute; blood pressure of 43/22mmHg; temperature of 35.2ºC; and respiratory rate of 44 breaths/minute. Pulse oximetry waveform was initially undetectable. On initial exam we noted markedly but symmetrically diminished lung aeration, a tensely distended abdomen and mottled skin with cyanosis. When we attempted supplemental ventilation with bag valve mask we noted high airway pressures. Concurrent with obtaining intravenous access and placing the patient on continuous monitoring, we obtained portable supine chest (Figure 1) and left lateral decubitus abdominal (Figure 2) radiographs.

**Figure 3:**
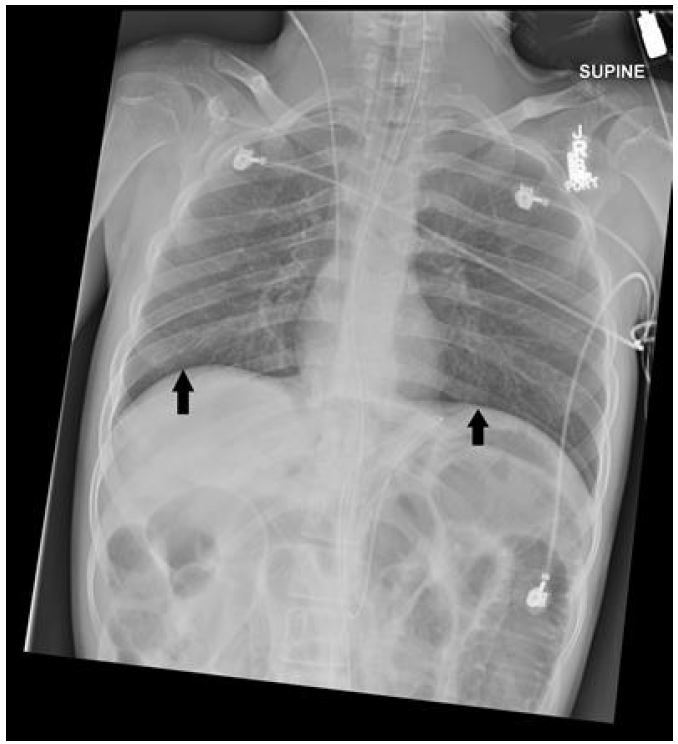
Chest x-ray performed after needle decompression of abdomen demonstrating improved diaphragmatic excursion (black arrows).

After noting massive pneumoperitoneum and elevation of the diaphragm we suspected tension pneumoperitoneum (TP) as the cause of shock. We positioned the patient in the right lateral decubitus position and placed three 14-gauge needles through the left abdominal wall, just lateral to the rectus musculature, and we advanced each needle until a rush of air was heard. As air was evacuated the patient’s abdominal distension visibly resolved. We soon noted decreased airway resistance and improved aeration. Within minutes the patient’s blood pressure improved to 93/47mmHg, and his pulse oximetry waveform became detectable at 93%. A repeat chest radiograph showed markedly improved pneumoperitoneum and diaphragmatic excursion with persistently dilated loops of bowel (Figure 3).

The patient was then taken to the operating room where he was found to have purulent peritonitis, and a 1.5cm cecal perforation was identified and repaired. A large rectal scybalum was also noted, and as there was no other bowel pathology identified during laparotomy, the cause of our patient’s bowel perforation was determined to be obstipation. We suspect that his preexisting communication difficulties caused a delay in presentation, allowing his constipation to progress to bowel perforation.

## DISCUSSION

Pneumoperitoneum is a radiographic term defined as free air within the peritoneal cavity. This finding usually suggests a perforated viscous, but up to 15% of cases occur in the absence of perforation.^1^ Pneumoperitoneum can occur post-procedurally or from passage of air from thoracic, abdominal, gynecologic or idiopathic sources (Table). Post-procedural pneumoperitoneum can follow percutaneous tube placement, laparotomy, laparoscopy and endoscopy. After laparoscopy air is expected to be visible on imaging for one week but may last up to four weeks.^2^ Approximately 0.1% of endoscopic procedures will result in pneumoperitoneum.^3^ The majority of these cases are the result of microperforation and do not result in peritonitis. In thoracic sources high intrathoracic pressure leads to introduction of air into the peritoneum via either diaphragmatic defects or perivascular connective tissue, allowing the mediastinum to communicate with the peritoneum. Pneumoperitoneum from abdominal sources occur through perforation of a hollow viscous, gas-forming bacterial infection (abscess or peritonitis) or rupture of gas-filled cysts within the alimentary tract wall (pneumatosis cystoides). Gynecologic sources of pneumoperitoneum are a result of the anatomic communication between the fallopian tubes and the peritoneal cavity and can occur under any circumstances that cause the pressure of air within the gynecologic organs to exceed the intraabdominal pressure (IAP) (mean IAP is between 16 and 20mmHg with a maximum of 25.5mmHg in non-pathologic states).^4^ The rate of pneumoperitoneum caused by processes that do not require surgical intervention has been estimated at 10%, so it is helpful to divide pneumoperitoneum into surgical and non-surgical categories.^5^ A method has been proposed to identify cases of pneumoperitoneum that do not require surgical intervention. If all of the following criteria are met it may be reasonable to consider non-surgical management: pneumoperitoneum is identified incidentally, there is a benign alternative explanation for the presence of air, there is no free fluid in the abdomen, and there are no signs of peritonitis or sepsis.^6^ While this method has not been prospectively validated, it is reasonable to consider managing with advanced imaging and serial examinations in these potentially non-surgical cases.

TP is an extreme form of pneumoperitoneum that occurs when intraabdominal free air reaches pressures high enough to impede venous return to the heart and to inhibit diaphragmatic excursion. Diminished venous return leads to decreased diastolic filling thereby decreasing cardiac output. This manifests as hypotension and decreased systemic perfusion. Inhibition of diaphragmatic excursion decreases tidal volume. If the patient is unable to compensate with an increased respiratory rate this will result in decreased minute ventilation and manifest as hypercarbic respiratory distress. If not diagnosed and treated promptly, TP will rapidly lead to death.

TP was first described in the medical literature by Oberst in 1917 when a grenade explosion resulted in gastric perforation.^7^ TP has since been reported as a complication of bowel perforation secondary to endoscopy, peptic ulcer disease and bowel obstruction.^8^–^12^ It is theorized that TP results from viscous perforation when overlying omental fat acts as a one-way valve, allowing gas to reach high pressures in the peritoneal cavity. It appears that barotrauma-induced TP without any viscous perforation is particularly rare, but this has also been reported.^13^ Of particular interest to emergency physicians are cases of TP following positive pressure ventilation during cardiopulmonary resuscitation with associated gastric rupture.^14^,^15^ Because the air is extraluminal, a gastric tube will not improve ventilation in these cases. Upon our review of the literature, we were unable to identify any previously reported cases of TP caused by constipation. We suspect that our patient’s history of MD, combined with his non-mobile and non-verbal status resulted in this novel presentation of TP. MD is known to be associated with increased colonic transit times, resulting not only from direct damage to smooth muscle, but also due to immobility and weak abdominal musculature contributing to constipation.^16^,^17^ Additionally, our patient was non-verbal. His inability to communicate his symptoms allowed his condition to progress unnoticed until it resulted in spontaneous perforation of his colon, a condition more commonly described in elderly patients with constipation.^18^ His non-verbal status further allowed this catastrophe to go unnoticed until it led to TP and its associated respiratory distress, the symptom that prompted his mother to bring him to the ED for evaluation.

The diagnosis of TP should be suspected in patients who present with respiratory distress associated with abdominal distension. Physical exam findings reveal high airway pressures, a tensely distended abdomen and poor systemic perfusion in TP. This diagnosis can be supported by chest and abdominal radiographs showing low lung volumes and a large amount of intraperitoneal free air. Sometimes the liver can also be observed to be inferiorly and medially displaced (the “saddle bag” sign). Initial treatment of TP consists of percutaneous needle decompression. The vast majority of reported cases of TP are associated with a perforated viscous. These patients should be emergently transferred to the operating room for diagnostic and therapeutic laparotomy unless there is highly compelling evidence to suggest that no such perforation has occurred.

## CONCLUSION

TP should be included in the differential diagnosis when assessing undifferentiated patients presenting to the ED with acute hemodynamic compromise. These patients present with hypotension, respiratory distress, a tensely distended abdomen and high inspiratory pressures. The diagnosis of TP can be supported by portable radiographs, and it can be confirmed with diagnostic and therapeutic needle decompression of the abdomen.

## Figures and Tables

**Table:** Causes of pneumoperitoneum.

Category	Cause
Abdominal	Anastomotic leak
	Collagen vascular diseases
	Diverticulosis (jejunal or sigmoid)
	Perforation of hollow viscus
	Pneumatosis cystoides intestinalis
	Rupture of intra-abdominal abscess
Gynecologic	Coitus
	Knee-chest exercise
	Pelvic inflammatory diseases
	Post-partum exercise
	Vaginal insufflation
	Vaginal douching
Thoracic	Adenotonsillectomy
	Asthma
	Barotrauma/thoracic trauma
	Bleb rupture (spontaneous)
	Bronchopulmonary fistula
	Bronchoscopy
	Cardiopulmonary resuscitation (can occur from blunt trauma, positive pressure ventilation or both)
	Pneumothorax/pneumomediastinum
	Positive end-expiratory pressure ventilation
	Pulmonary tuberculosis
	Severe coughing
Post-procedural	Laparoscopy/laparotomy
	Endoscopy
	Gynecologic examination procedures
	Postpolypectomy syndrome
	Percutaneous enteric tube placement/displacement
	Peritoneal dialysis
Other	Gas forming bacterial infection
	Idiopathic
